# Supplementary material for: Increased AID results in mutations at the CRLF2 locus implicated in Latin American ALL health disparities
Source: Nat Commun. 2024 Jul 27;15:6331. doi: 10.1038/s41467-024-50537-0 (PMC11283463; doi:10.1038/s41467-024-50537-0)
Supplement: Supplementary file 1 — Supplementary Information [file 41467_2024_50537_MOESM1_ESM.pdf]

SUPPLEMENTAL INFORMATION

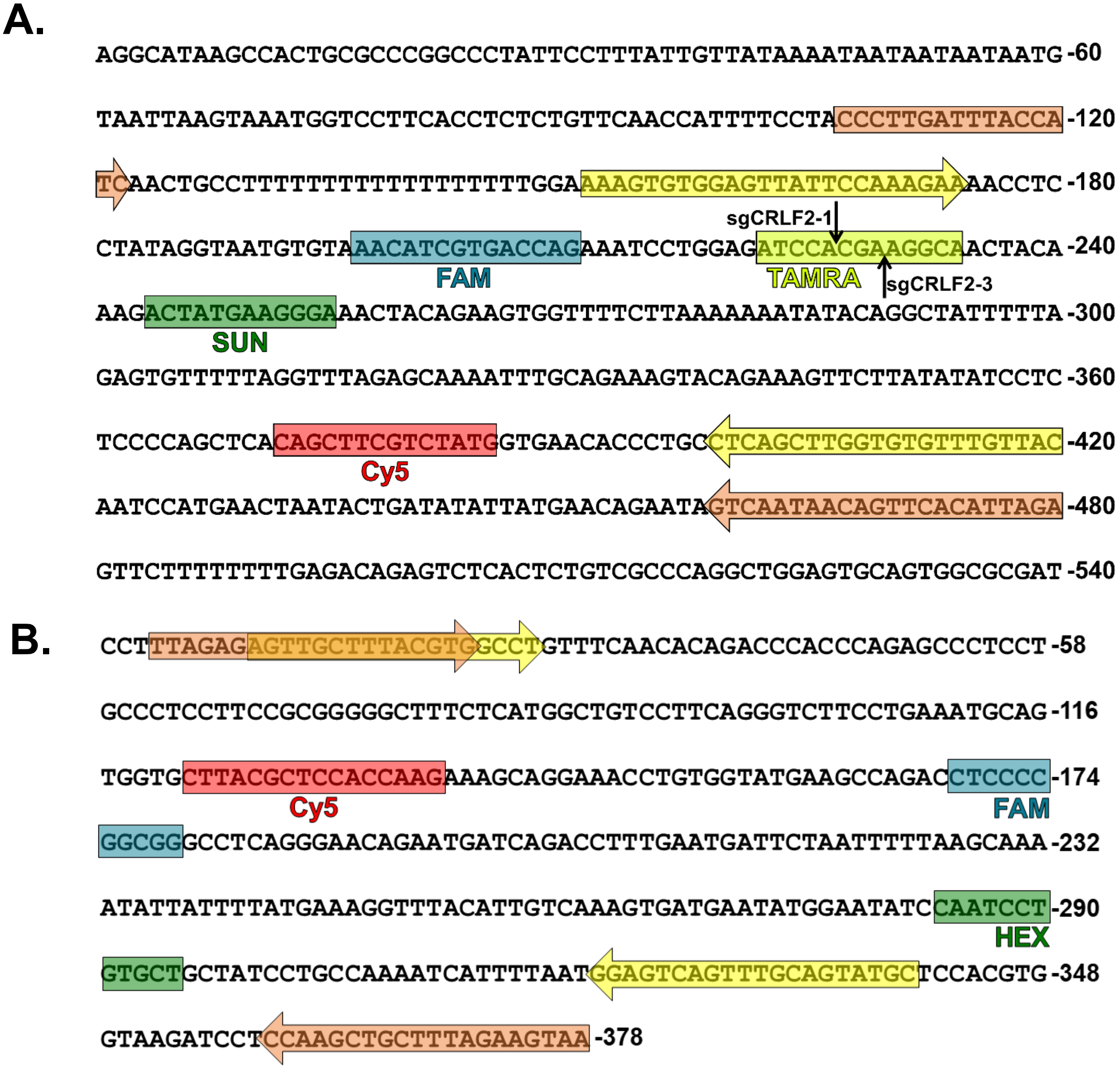

Supplementary Figure 1. DNA sequences of the ABCs at *CRLF2* and *BCL2* (A) *CRLF2* ABC sequence, Chr X: 1228310-1228969 (same coordinates for Chr Y). Orange arrows indicate sequences of amplicon primers for AmpSeq and yellow arrows are amplicon primers for dPCR. Blue, yellow, green, and red boxes indicate sequences where the FAM, TAMRA, SUN, and Cy5 drop-off probes bind, respectively. Vertical arrows indicate cut sites targeted by Cas9 via sgCRLF2-1 and sgCRLF2-3. **(B)** *BCL2* ABC sequences, Chr 18: 63126040-63126870. Orange arrows indicate sequences of amplicon primers for AmpSeq and yellow arrows indicate sequences of amplicon primers for dPCR. Red, blue, and green boxes indicate sequences where the Cy5, FAM, and HEX drop-off probes bind, respectively. For simplicity, only one strand is shown, so the reverse amplicon primers are the reverse complement. For all exact sequences, see Supplemental Table 1. All chromosome coordinates are from the December

2013 GRch38/gh38 sequencing build. dPCR program: 96°C for 10 minutes (1x); 96°C for 10 seconds, 63°C for 20 seconds, 68°C for 30 seconds (40x).

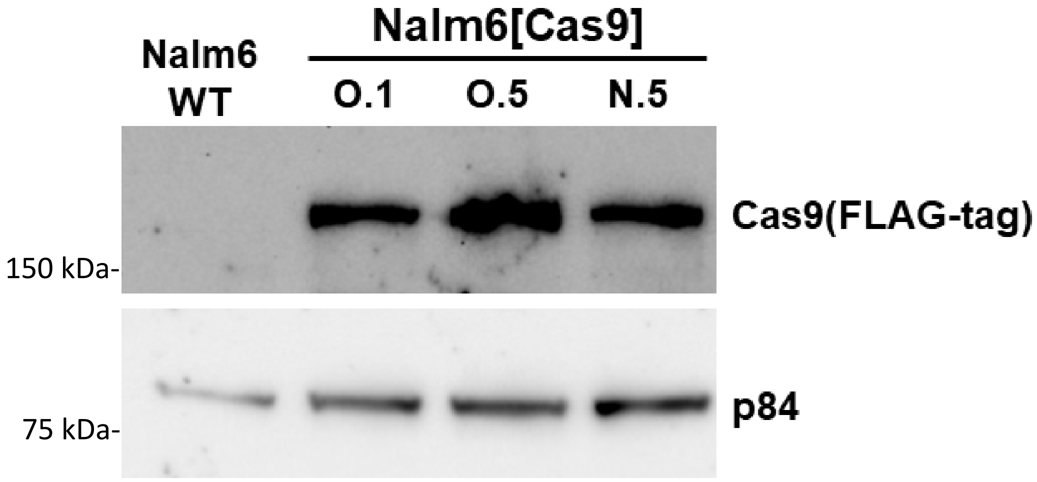

Supplementary Figure 2. Western blot showing protein abundance of Cas9 in Nalm6-Cas9 cells with a constitutively active Cas9 integrated via a lentiviral vector. The Cas9 and PuroR genes are expressed as a single transcript with the P2A self-cleaving peptide separating the proteins following translation. Maintaining cells under puromycin selection ensures the Cas9 remains active. Source data are provided as a Source Data file.

A.

	No sgRNA	sgIGHM1	sgIGHM1 + sgBCL2
**JoinT** (translocations)	223	2,532	464
**JoinT** (breaksite)	60	81,738	13,890
**JoinT** (total)	283	84,270	14,354

	sgCRLF2	sgIGHM6	sgIGHM6 + sgCRLF2
**JoinT** (translocations)	97	924	557
**JoinT** (breaksite)	64	392,873	358,144
**JoinT** (total)	161	393,797	358,701

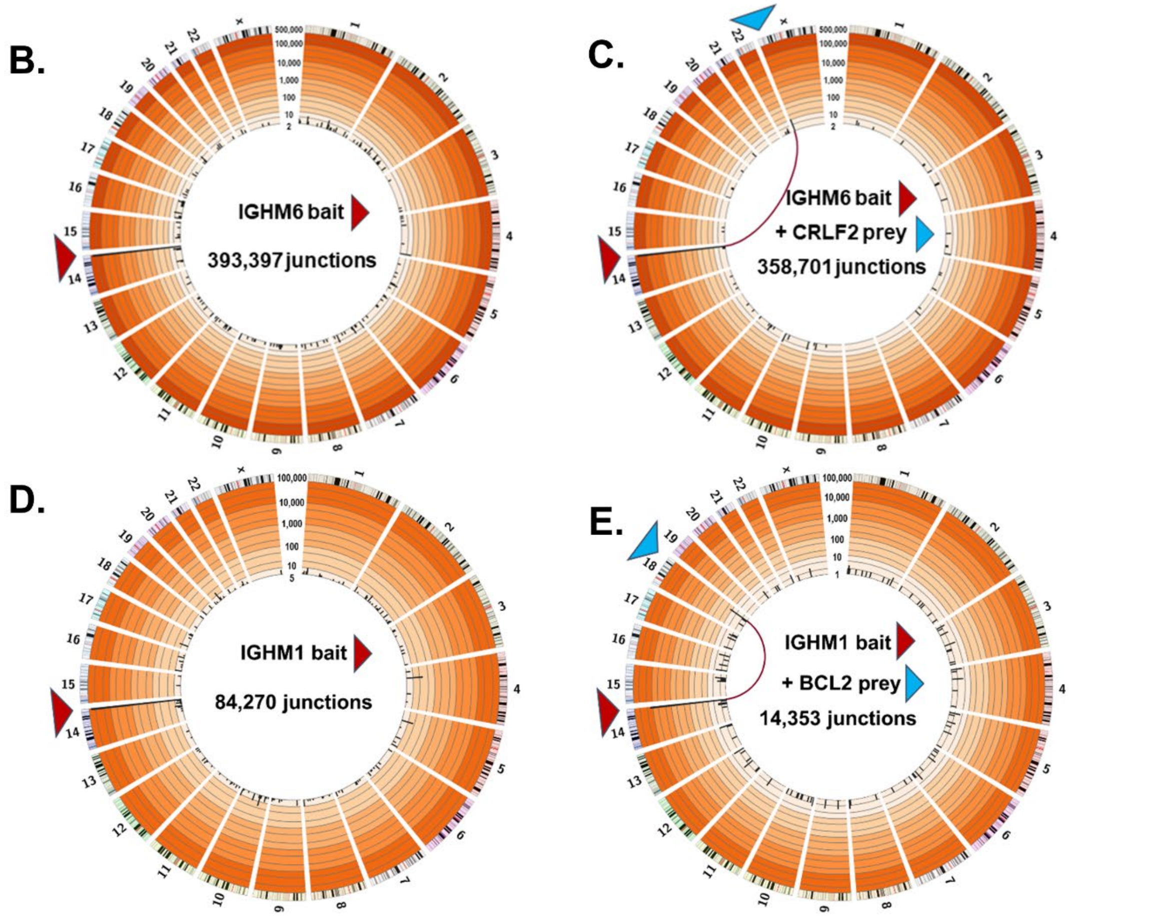

Supplementary Figure 3. HTGTS-JoinT-seq was mapped translocations following Cas9-induced DBSs at IGH, CRLF2 and BCL2. (A) Table listing the breakdown of junctions with or without Cas9 bait DSBs (sgIGH-1 or sgIGH-6) and ABC prey DSBs (sgCRLF2-1 or sgBCL2). **(B)** Circos plots demonstrating breakpoint junctions and/or translocations in Nalm6-Cas9 cells transfected with sgIGH-6 (bait) alone or (**C**) co-transfected with sgIGH-6 and sgCRLF2-1. (**D**) Circos plots demonstrating breakpoint junctions and/or translocations in Nalm6-Cas9 cells transfected with sgIGH-1 alone or (**E**) co-transfected with sgIGH-1 and sgBCL2. For B-D, a red arrow indicates the *IGH* bait DSB and a blue arrow indicates the *CRLF2* or *BCL2* prey DSB. Total number of breakpoint junctions measured is indicated. Connecting red lines show the bait/prey translocation formed.

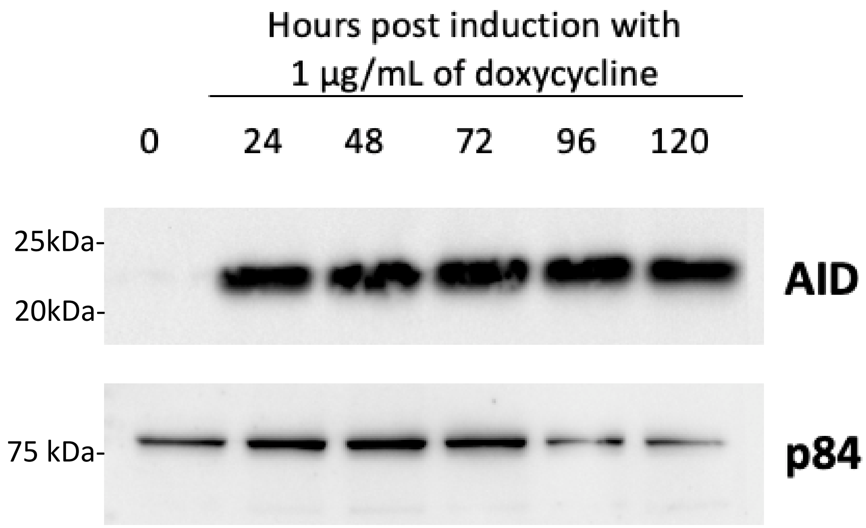

Supplementary Figure 4. Western blot showing protein abundance of AID in Nalm6-AID cells with dox-inducible AID cassette integrated via a lentiviral vector. The AID and G418R genes are expressed as a single transcript with the P2A self-cleaving peptide separating the proteins following translation. Maintaining cells under G418 selection ensures the dox-inducible AID cassette remains present. The experiment was repeated once with similar results. Source data are provided as a Source Data file.

Supplementary Table 1. Blood Samples Collected from Healthy Donors

Sample	Age Range	Sex	Ethnicity
H-H/L-1[a]	20-50	F	Hispanic
H-H/L-2	50-80	F	Hispanic
H-H/L-3	20-50	M	Hispanic
H-H/L-4	20-50	F	Hispanic
H-H/L-5	20-50	F	Hispanic
H-W-1[b]	50-80	F	White
H-W-2	20-50	F	White
H-W-3	20-50	M	White
H-W-4	50-80	F	White
H-A-1[c]	20-50	F	Asian
H-A-2	20-50	F	Asian

[a]H-H/L = Healthy Hispanic/Latino

[b]H-W = Healthy White

[c]H-A = Healthy Asian

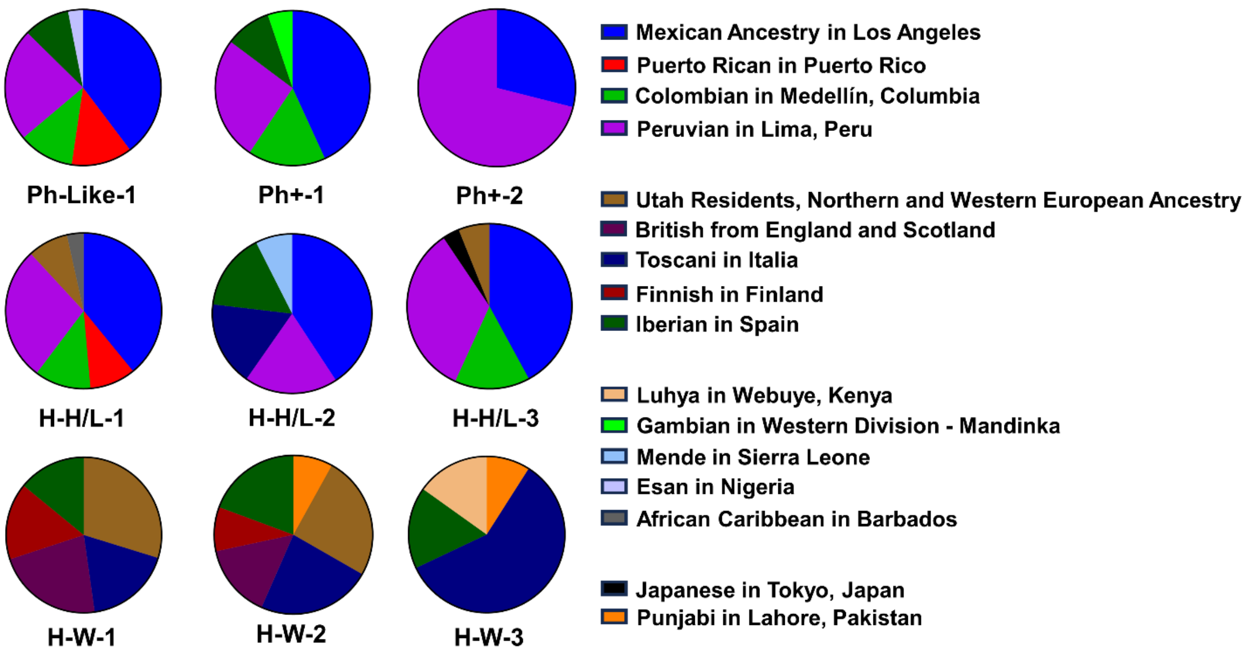

Supplementary Figure 5. Genetic admixture analysis of select human samples used in this study. An Illumina Global Diversity Array was used to detect single nucleotide polymorphisms (SNPs) that correlate with genetic ancestry. These SNPs were then cross referenced with Hispanic and Latino samples using the 1000 Genome reference map to determine the admixture probability for each subpopulation.

A. **Pathway Enrichment of DEGs from Ph-Like Cohort**

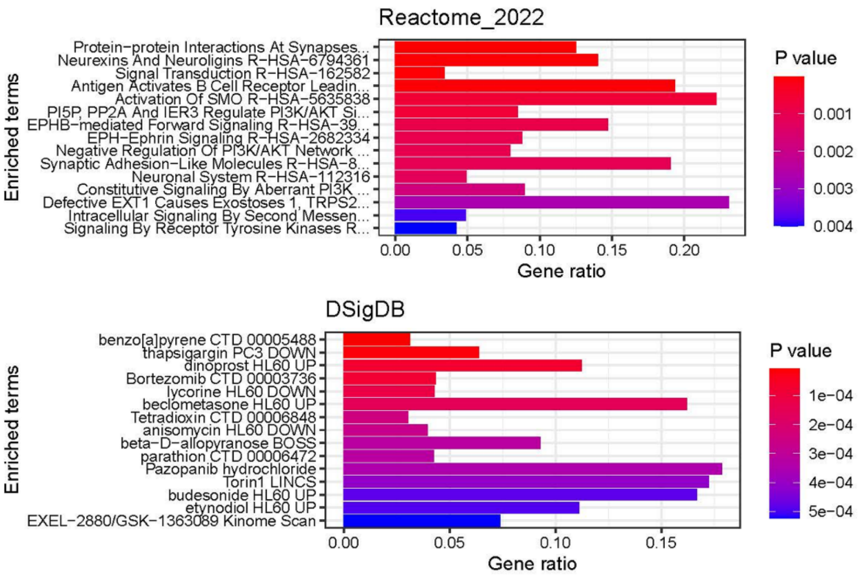

B. **Pathway Enrichment of DEGs from Ph+ Cohort**

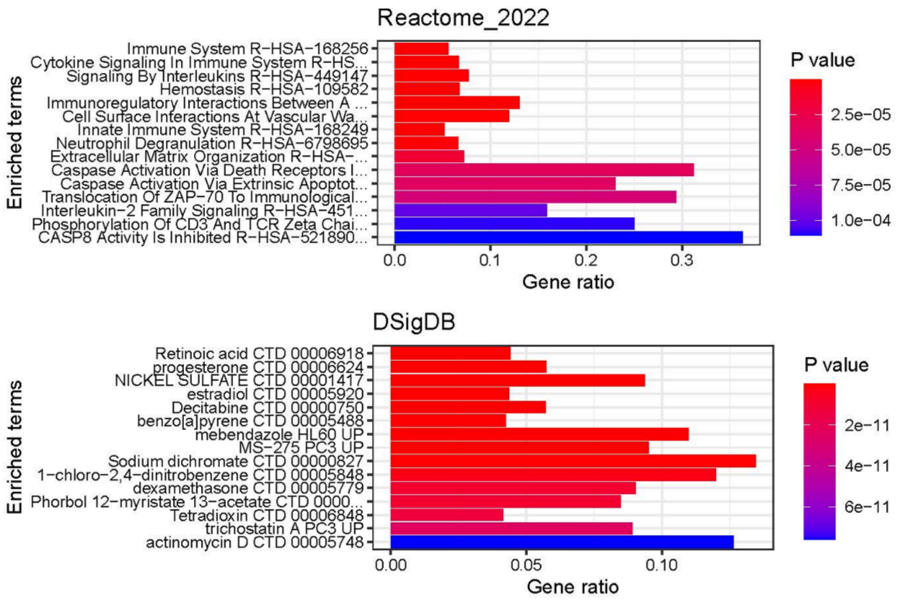

Supplementary Figure 6. Pathway enrichment analysis comparing Ph-like and Ph+ datasets. Gene Set Enrichment Analysis (GSEA) was performed using the pathway databases Reactome, and Drug Signatures Database (DSigDB) showing pathway enrichment from the **(A)** patients Ph-like -1 and Ph-like-2 and **(B)** patients Ph+-1 and Ph+-2.

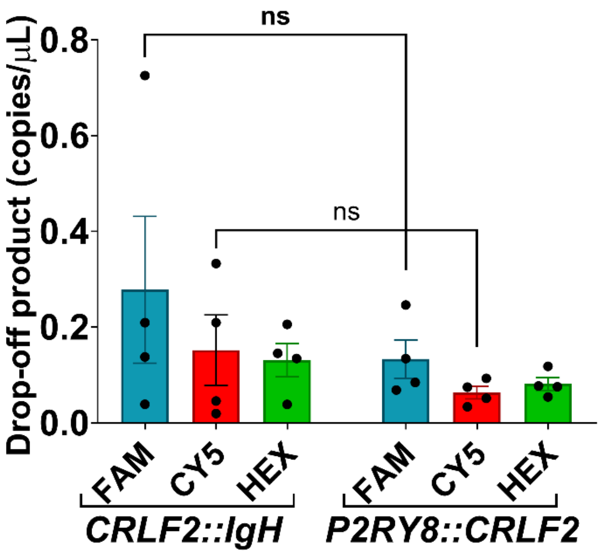

Supplementary Figure 7. Comparison of quantified drop-off product for the FAM, Cy5 and HEX probes associated with the *BCL2* ABC between Hispanic Ph-Like ALL patients with *CRLF2::IGH* or *P2RY8::CRLF2* rearrangements. Each data point represents the mean from at least six technical replicates using gDNA from an individual patient. Here, all 4 Ph-like ALL patients with *CRLF2::IGH* are shown whereas in Fig. 5A, only 3 are shown as Ph-like-4 is displayed separately in Fig. 5B. Drop-off product for *CRLF2::IgH* is plotted as mean values ± SEM from 4 individual diagnosed patients. Drop-off product for *P2RY8::CRLF2* is plotted as mean values ± SEM from 4 individual diagnosed patients. Not significant (ns) by results of an Unpaired *t* test (p=0.3967 for FAM, p=0.2801 for Cy5) (n=4 for *CRLF2::IgH*, n=4 for *P2RY8::CRLF2*). Source data are provided as a Source Data file.

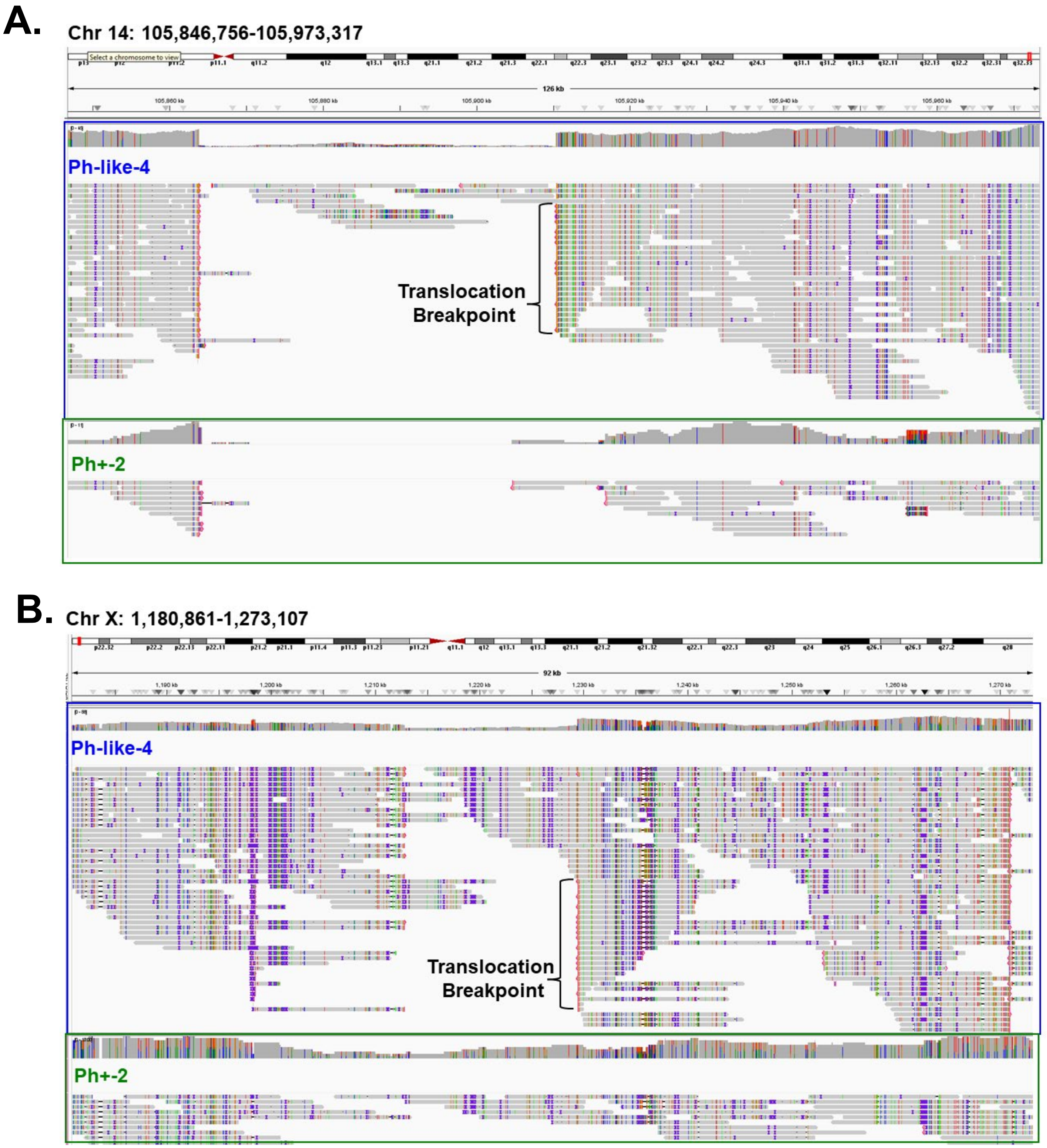

Supplementary Figure 8. Alignment of long-read sequencing data. Screenshot of **(A)** Chr. 14 and **(B)** Chr. X from the Integrative Genomics Viewer (IGV) displaying data long read sequencing of patients Ph-like-4 and Ph+-2. BAM files were loaded and reads aligned to the hg38 human reference genome. Translocation breakpoints for Ph-like-4 are indicated where a hybrid read between chromosomes 14 and

X was detected. Both patients are male but reads from the shared PAR between chromosomes X and Y are mapped to the X chromosome.

Supplementary Table 2. Chromosome Instability in Ph-like-4 and Ph+-2 Patients

	hg38 Genomic Coordinates	
Locus	Ph-Like-4	Ph+-2
IGH	chr14:105,856,341-105,873,004	chr14:105,856,341-105,873,004
CRLF2	chrX:1,206,101-1,230,261	No Structural Variant
BCR	chr22:23,282,759-23,300,199	chr22:23,282,759-23,300,199
ABL1	No Structural Variant	chr9:130,839,992-130,845,657
IKZF1	chr7:50,333,391-50,338,511	No Structural Variant
	chr7:50,297,986-50,323,897	No Structural Variant
JAK2	chr9:5,085,952-5,104,396	No Structural Variant
	chr9:5,016,355-5,034,799	No Structural Variant
CDKN2A	chr9:21,966,752-21,996,324	No Structural Variant
TCR	chr14:21,942,101-21,956,635	No Structural Variant
