# Supplementary material for: Increased AID results in mutations at the CRLF2 locus implicated in Latin American ALL health disparities
Source: Nat Commun. 2024 Jul 27;15:6331. doi: 10.1038/s41467-024-50537-0 (PMC11283463; doi:10.1038/s41467-024-50537-0)
Supplement: Supplementary file 3 — Description of Additional Supplementary Files [file 41467_2024_50537_MOESM3_ESM.pdf]

Description of Additional Supplementary Files

File Name: Supplementary Data 1
Description: Full list of probe and primer sequences

File Name: Supplementary Data 2
Description: Complete list of the differentially expressed genes (DEG's) shown in figure 4.
