# Supplementary material for: Increased AID results in mutations at the CRLF2 locus implicated in Latin American ALL health disparities
Source: Nat Commun. 2024 Jul 27;15:6331. doi: 10.1038/s41467-024-50537-0 (PMC11283463; doi:10.1038/s41467-024-50537-0)
Supplement: Supplementary file 6 — Reporting Summary [file 41467_2024_50537_MOESM6_ESM.pdf]

nature portfolio

Corresponding author(s): Nicholas R Pannunzio

Last updated by author(s): Jun 11, 2024

Reporting Summary

Nature Portfolio wishes to improve the reproducibility of the work that we publish. This form provides structure for consistency and transparency in reporting. For further information on Nature Portfolio policies, see our Editorial Policies and the Editorial Policy Checklist.

Statistics

For all statistical analyses, confirm that the following items are present in the figure legend, table legend, main text, or Methods section.

n/a	Confirmed	
☐	☒	The exact sample size (*n*) for each experimental group/condition, given as a discrete number and unit of measurement
☐	☒	A statement on whether measurements were taken from distinct samples or whether the same sample was measured repeatedly
☐	☒	The statistical test(s) used AND whether they are one- or two-sided<br>*Only common tests should be described solely by name; describe more complex techniques in the Methods section.*
☐	☒	A description of all covariates tested
☐	☒	A description of any assumptions or corrections, such as tests of normality and adjustment for multiple comparisons
☐	☒	A full description of the statistical parameters including central tendency (e.g. means) or other basic estimates (e.g. regression coefficient) AND variation (e.g. standard deviation) or associated estimates of uncertainty (e.g. confidence intervals)
☐	☒	For null hypothesis testing, the test statistic (e.g. *F*, *t*, *r*) with confidence intervals, effect sizes, degrees of freedom and *P* value noted<br>*Give P values as exact values whenever suitable.*
☒	☐	For Bayesian analysis, information on the choice of priors and Markov chain Monte Carlo settings
☒	☐	For hierarchical and complex designs, identification of the appropriate level for tests and full reporting of outcomes
☒	☐	Estimates of effect sizes (e.g. Cohen's *d*, Pearson's *r*), indicating how they were calculated

Our web collection on statistics for biologists contains articles on many of the points above.

Software and code

Policy information about availability of computer code

Data collection	ThermoFisher Quantstudio Absolute Q Software (v6.3), FASTQC to check FASTQ files (v0.11.9), STAR aligner (v2.7.10), GATK (v4.5.0.0), DESeq2 for differential gene expression (v1.44.0), cuteSV (v2.1.1).
Data analysis	GraphPad Prism (10.2.2), Integrative Genomics Viewer (IGV) (v2.17.2.01) for aligning and mapping DNA sequencing reads.

For manuscripts utilizing custom algorithms or software that are central to the research but not yet described in published literature, software must be made available to editors and reviewers. We strongly encourage code deposition in a community repository (e.g. GitHub). See the Nature Portfolio guidelines for submitting code & software for further information.

Data

Policy information about availability of data

All manuscripts must include a data availability statement. This statement should provide the following information, where applicable:

- Accession codes, unique identifiers, or web links for publicly available datasets
- A description of any restrictions on data availability
- For clinical datasets or third party data, please ensure that the statement adheres to our policy

HTGTS data were deposited into the Gene Expression Omnibus database under accession number GSE243667. RNA-seq data has been deposited in Sequence Read Archive (SRA, Bioproject PRJNA31257).

Research involving human participants, their data, or biological material

Policy information about studies with human participants or human data. See also policy information about sex, gender (identity/presentation), and sexual orientation and race, ethnicity and racism.

Reporting on sex and gender	All data on sex is self-reported. Sex was not a factor in the study design and the outcomes to not suggest that sex is a factor.
Reporting on race, ethnicity, or other socially relevant groupings	Race and ethnicity data are from self-reporting. Hispanic, non-Hispanic White, and Asian is used as this was their self-reported ethnicity listed in the patient file. "Hispanic" in this sense can refer to Latin Americans and those of European/ Spanish decent. Admixture analysis was done on a subset of patients and showed that several of the patients that self-identify as Hispanic have genetic similarities to populations in Mexico, Central, and South America. Since admixture analysis was not performed on all patients, we use the self-reported "Hispanic" term most often to refer to this group.
Population characteristics	Recruitment and consent if in populations over 18 upon cancer diagnosis and prior to treatment.
Recruitment	Study staff will contact the inpatient UCI leukemia service "Team L" each morning to ask if there have been any new known/ suspected ALL cases in the past day. If the answer is yes then study staff will approach these new potential participants in person or by phone (in person contact may not be possible due to COVID restrictions). Study staff will introduce themselves as part of a UCI research team and will explain the purpose of the study and what the study entails. The participant will then be given the opportunity to sign a consent form (or do a verbal consent depending on COVID restrictions).<br>Note: this is the protocol for ALL patients, normal controls will not be actively recruited by this method, deidentified normal controls will be collected from ICTS normal control resource.
Ethics oversight	University of California, Irvine Institutional Review Board

Note that full information on the approval of the study protocol must also be provided in the manuscript.

Field-specific reporting

Please select the one below that is the best fit for your research. If you are not sure, read the appropriate sections before making your selection.

☒ Life sciences ☐ Behavioural & social sciences ☐ Ecological, evolutionary & environmental sciences

For a reference copy of the document with all sections, see nature.com/documents/nr-reporting-summary-flat.pdf

Life sciences study design

All studies must disclose on these points even when the disclosure is negative.

Sample size	No sample size calculations were reported. Due to the rarity of Ph-like ALL, we obtained as many Ph-like ALL samples as possible. An overall goal for our study is to eventually have a biobank of up to 50 samples.
Data exclusions	No data was excluded from the analysis.
Replication	For experiments with human cell lines, at least 6 biological replicates were performed. For human samples, only one DNA samples was collected, so experiments using that sample were repeated at least 6 times to be results were consistent. Westerns and sequencing was performed in triplicate to confirm consistent results.
Randomization	Randomization was not relevant to our study.
Blinding	Blinding was not relevant to our study.

Reporting for specific materials, systems and methods

We require information from authors about some types of materials, experimental systems and methods used in many studies. Here, indicate whether each material, system or method listed is relevant to your study. If you are not sure if a list item applies to your research, read the appropriate section before selecting a response.

Materials & experimental systems

n/a	Involved in the study
☐	☒ Antibodies
☐	☒ Eukaryotic cell lines
☒	☐ Palaeontology and archaeology
☒	☐ Animals and other organisms
☒	☐ Clinical data
☒	☐ Dual use research of concern
☒	☐ Plants

Methods

n/a	Involved in the study
☒	☐ ChIP-seq
☒	☐ Flow cytometry
☒	☐ MRI-based neuroimaging

Antibodies

Antibodies used

AID (L7E7) (Cell Signaling, 4975); FLAG M2 (Sigma-Aldrich, A2220); p84 (GeneTex, GTX70220)

Validation

AID antibody from Cell Signaling was validated by Western blot analysis of extracts from P3HR-1 and Ramos cells using AID (L7E7) Mouse mAb and Western blot analysis of immunoprecipitates from HeLa cell extracts overexpressing myc-tagged AID. Expression of myc-AID was confirmed (lane 1). Immunoprecipitations using cell lysates with or without expression of myc-AID were performed, using AID (L7E7) Mouse mAb #4975, Myc-Tag Antibody #2272, mouse IgG or rabbit IgG. Immunoblot was performed using Myc-Tag Antibody #2272. FLAG M2 was validated as described in previous citations (PMIDs:17274760, 15044443, 18227151). p84 was validated by the manufacture, GTX70220 IP Image:p84 antibody [5E10] immunoprecipitates p84 protein in IP experiments. IP Sample: HepG2 whole cel lysate/extract A : 30 µg whole cel lysate/extract of p84 protein expressing HepG2 cels B : Control with 3 µg of pre-immune mouse IgG C : Immunoprecipitation of p84 by 3 µg of p84 antibody [5E10] (GTX70220) 7.5% SDS-PAGE The immunoprecipitated p84 protein was detected by p84 antibody [5E10] (GTX70220) diluted at 1 : 1000. EasyBlot anti-rabbit IgG (HRP) (GTX221667-01) was used as a secondary reagent.

Eukaryotic cell lines

Policy information about cell lines and Sex and Gender in Research

Cell line source(s)

Nalm6 (CRL-3273) and Reh (CRL-8286)cell lines were purchased from the American Type Culture Collection (ATCC, Manassas, Virginia)

Authentication

Cell lines were authenticated by ATCC.

Mycoplasma contamination

Cell lines were confirmed to be mycoplasma-free by ATCC and regularly test negative.

Commonly misidentified lines
(See ICLAC register)

N/A

Plants

Seed stocks

Report on the source of all seed stocks or other plant material used. If applicable, state the seed stock centre and catalogue number. If plant specimens were collected from the field, describe the collection location, date and sampling procedures.

Novel plant genotypes

Describe the methods by which all novel plant genotypes were produced. This includes those generated by transgenic approaches, gene editing, chemical/radiation-based mutagenesis and hybridization. For transgenic lines, describe the transformation method, the number of independent lines analyzed and the generation upon which experiments were performed. For gene-edited lines, describe the editor used, the endogenous sequence targeted for editing, the targeting guide RNA sequence (if applicable) and how the editor was applied.

Authentication

Describe any authentication procedures for each seed stock used or novel genotype generated. Describe any experiments used to assess the effect of a mutation and, where applicable, how potential secondary effects (e.g. second site T-DNA insertions, mosiacism, off-target gene editing) were examined.
